# Associations of the atherogenic index of plasma with insulin resistance and inflammation

**DOI:** 10.1590/1806-9282.20240991

**Published:** 2024-12-02

**Authors:** Yasin Altun, Halime Dilber Balcı, Nilay Çom Aybal

**Affiliations:** 1 Niğde Omer Halisdemir University, Faculty of Medicine, Department of Family Medicine – Niğde, Turkey.; 2 Niğde Omer Halisdemir University Research and Training Hospital – Niğde, Turkey.; 3 Yalova University, Faculty of Medicine, Department of Family Medicine – Yalova, Turkey.

**Keywords:** Inflammation, Insulin resistance, Atherogenesis

## Abstract

**OBJECTIVE:**

This study aimed to investigate the association between the atherogenic index of plasma and insulin resistance, while also evaluating atherogenic index of plasma's prognostic value in inflammation by examining its correlation with platelet/lymphocyte ratio and neutrophil/lymphocyte ratio.

**METHODS:**

Data collection involved accessing records from January 2022 to December 2022, with analyses including calculation of atherogenic index of plasma and homeostatic model assessment index.

**RESULTS:**

Data from 510 patients (mean age: 48.90±18.71 years; 69.4% female) showed a mean atherogenic index of plasma of 0.40±0.28, with 69% at high risk. Significant positive correlations were found between atherogenic index of plasma and age, lymphocyte, neutrophil, C-reactive protein, glucose, insulin, and homeostatic model assessment-insulin resistance, while the correlation with platelet/lymphocyte ratio was significantly negative.

**CONCLUSION:**

Atherogenic index of plasma presents as a promising predictor for insulin resistance, demonstrating robust associations with inflammation, thus offering valuable clinical insights and potential for early risk assessment and management strategies.

## INTRODUCTION

Insulin resistance (IR) is characterized by the reduced body's cells’ response to insulin and is a significant risk factor for cardiovascular disease (CVD), independent of diabetes. Beyond glucose metabolism, IR disrupts lipid metabolism, raising triglycerides (TGs) and low-density lipoprotein cholesterol (LDL-C), which are linked to atherosclerosis and CVD. IR often coexists with systemic inflammation and oxidative stress, worsening metabolic dysfunction and chronic diseases. Atherosclerosis, driven by lipid-induced immune-inflammatory processes, leads to CVD^1^. Inflammation significantly contributes to atherosclerosis formation and progression, increasing the risk of dyslipidemia^2^. Dyslipidemia, characterized by high LDL-C, total cholesterol, TGs, and low high-density lipoprotein cholesterol (HDL-C), contributes to atherosclerosis development^3^.

The atherogenic index of plasma (AIP) is determined by the logarithm of TG/HDL-C and is recognized as a strong predictor for CVD^4^. AIP assessment not only indicates coronary atherosclerosis and cardiovascular risk but is also an enhanced and effective measure of cardiovascular risk factors^5^. According to several studies, AIP reflects CVD risk better than other atherogenic indexes or lipoprotein cholesterol parameters alone^6^.

Type 2 diabetes mellitus (T2DM) is a major CVD risk factor, with higher CVD mortality compared to nondiabetic individuals^7^. IR, often linked to T2DM and measured by the homeostatic model assessment (HOMA-IR) with values above 2.5 indicating IR, heightens CVD risk^8^. Sharma et al. found a strong link between elevated fasting blood glucose (FBG) and increased AIP^9^. In AIP, values of −0.3 to 0.1 indicate low CVD risk, 0.1–0.24 moderate risk, and above 0.24 high risk^10^.

According to various studies, both AIP and IR are closely associated with inflammation, with AIP reflecting cardiovascular risk driven by inflammatory processes^11,12^. Platelet, lymphocyte, and neutrophil counts are key prognostic markers in clinical practice. The platelet/lymphocyte (PLR) and neutrophil/lymphocyte (NLR) ratios are used as biomarkers for inflammatory conditions like CVD, cancer, and autoimmune diseases. Elevated NLR and PLR are linked to higher inflammation severity and are strong, independent predictors of mortality risk in patients with comorbidities^13^.

This study aimed to investigate the relationship between AIP and IR and to assess AIP's role in diagnosing and monitoring T2DM. In addition, we evaluated AIP's prognostic values for inflammation by examining its correlation with inflammatory markers like NLR, PLR, and C-reactive protein (CRP).

## METHODS

### Patients and design

Within the scope of the study, complete blood count, CRP, albumin, HDL-C, LDL-C, TGs, and FBG records of the patients who received service from the family medicine outpatient clinic between January 2022 and December 2022 were accessed by retrospective inquiry through the hospital information system. Age, gender, diagnoses, and test results were recorded on the data collection form. AIP and HOMA-IR were calculated from these data.

### Ethical approval

The study was approved by the Nigde University Research Ethics Committee (Approval No. 2023/41).

### Calculation of the parameters

#### Atherogenic index of plasma

Dobiásová et al. described AIP as a sensitive and potential index of the atherogenicity of lipoproteins. AIP is calculated as log^10^ (TG/HDL cholesterol)^5^.

#### Homeostatic model assessment-insulin resistance

HOMA-IR is an easier test to perform than other methods that can demonstrate both IR and β-cell functions. The calculation is performed by multiplying the insulin level in mIU/L by the glucose level in mg/dL and then dividing the result by 405^14^.

### Data analysis

Data analysis was done in the SPSS v25 package program. The normality of continuous data was assessed using the Kolmogorov-Smirnov test. Independent-samples t-test was used in independent groups to compare two groups in continuous data. Relationships were examined with Spearman correlation analysis since parametric test conditions were not met. Binary logistic regression with Forward:LR was used to examine the factors affecting the presence of a high-risk AIP. Variables with p<0.25 in univariate analysis and variables that were thought to clinically affect the dependent variable were included in the model. The fit of the model was evaluated with the Hosmer-Lemeshow test and its descriptiveness with Nagelkerke R2. All analyses were conducted using a two-tailed approach, and significance was defined as p<0.05.

In the post hoc power analysis performed after the study, the effect size between the presence of IR and AIP was found to be 0.473 and the power of the study was calculated as 99%.

## RESULTS

The data of 510 patients were examined. The mean age of the participants was 48.90±18.71 years, and 69.4% of them were women. The mean AIP of the participants was 0.40±0.28, with 69% classified as high risk for AIP. In addition, 50.8% of the participants had IR (HOMA-IR≥2.5). The mean values of the participants’ biochemical parameters are shown in Table 1.

**Table 1:** Distribution of biochemical parameters of patients.

	Mean±standard deviation	Median (minimum–maximum)
Age (years)	48.90±18.71	49.50 (14–93)
NLR	2.03±1.04	1.78 (0.54–10.27)
PLR	126.62±50.05	119.13 (15.95–582.05)
Lymphocyte (10^3^/mcL)	2.30±0.73	2.28 (0.76–6)
Platelet (10^3^/mcL)	269.78±71.11	267 (41–599)
Neutrophil (10^3^/mcL)	4.29±1.53	4.09 (1.20–14.79)
CRP (mg/L)	4.50±10.13	1.90 (0–120.50)
Glucose (mg/dL)	110.16±48.58	94 (65–441)
HDL (mg/dL)	49.40±12.53	48 (22–107)
Triglyceride (mg/dL)	140.08±88.90	115 (25–735)
Insulin (mIU/L)	15.76±29.91	10.40 (0.40–591)
AIP	0.40±0.28	0.39 (-0.62 to 1.34)
HOMA-IR	4.72±15.03	2.54 (0.22–321.04)
		n (%)
Gender	Female	354 (69.4%)
	Male	156 (30.6%)
AIP risk classification	Low risk (API<0.11)	76 (14.9%)
	Medium risk (API=0.11–0.24)	82 (16.1%)
	High risk (API>0.24)	352 (69%)
Presence of IR	No IR (HOMA-IR<2.5)	251 (49.2%)
	IR (HOMA-IR≥2.5)	259 (50.8%)

NLR: neutrophil/lymphocyte ratio; PLR: platelet/lymphocyte ratio; CRP: C-reactive protein; HDL: high-density lipoprotein; AIP: atherogenic index of plasma; HOMA-IR: homeostatic model assessment-insulin resistance; IR: insulin resistance.

Significant positive correlations exist between AIP and various factors, including age, lymphocyte, neutrophil, CRP, glucose, insulin, and HOMA-IR (p<0.05). Despite these results, the correlation between AIP and PLR was significantly negative (p<0.001). The AIP of the patients was also compared based on their gender and IR values. The results indicated significant differences in AIP among genders and IR values (p<0.001) (Table 2).

**Table 2:** Correlation of atherogenic index of plasma with other variables.

Variables	r	p*
Age	0.363	<0.001
NLR	-0.003	0.942
PLR	-0.223	<0.001
Lymphocyte	0.190	<0.001
Platelet	-0.081	0.068
Neutrophil	0.151	0.001
CRP	0.242	<0.001
Glucose	0.301	<0.001
Insulin	0.241	<0.001
HOMA-IR	0.330	<0.001
		p**
Gender	Female	<0.001
	Male	
Presence of IR	No IR (HOMA-IR<2.5)	<0.001
	IR (HOMA-IR≥2.5)	

* Spearman correlation test;

** Independent samples’ t-test. AIP: atherogenic index of plasma; NLR: neutrophil/lymphocyte ratio; PLR: platelet/lymphocyte ratio; CRP: C-reactive protein; HOMA-IR: homeostatic model assessment-insulin resistance; IR: insulin resistance.

Gender, age, lymphocyte, and IR had the highest explanatory values in the multivariate logistic regression model. Being male increased the likelihood of high-risk AIP by 2.97 times and IR by 2.23 times. Each unit increase in age and lymphocyte also increased the likelihood of high-risk AIP by 1.03 and 1.85 times, respectively (Table 3).

**Table 3:** Evaluation of factors affecting the status of having a high-risk atherogenic index of plasma.

Variables	OR (95%CI)	p
Gender (ref: Female)	2.97 (1.79–4.93)	<0.001
Age	1.03 (1.02–1.04)	<0.001
Lymphocyte	1.85 (1.34–2.56)	<0.001
Presence of IR (ref: no IR)	2.23 (1.45–3.42)	0.001

R²=0.26, −2 loglikelihood=526.34, Omnibus test:<0.001, Hosmer-Lemeshow test: 0.191. CI: confidence interval; OR: odds ratio; IR: insulin resistance.

## DISCUSSION

AIP, which comprehensively measures lipid levels, serves as a potent biomarker for dyslipidemia and atherosclerosis. Furthermore, it is considered indicative of coronary and metabolic syndromes^15^. Elevated AIP values are associated with coronary artery disease (CAD) and its risk factors, such as hyperlipidemia, metabolic syndrome, and hypertension^16^. In our study, the average AIP was 0.40, with 69% of the participants classified as high risk. Significant correlations were observed between AIP and both age and gender. Furthermore, the AIP values of males were higher than those of females, similar to the findings of a study conducted in the Czech Republic^10^. In a study by Cai et al., AIP was significantly positively associated with age^15^. Older age and male gender are considered at risk in terms of AIP values, indicating an increased risk of CVD. These results could have a meaningful impact on comprehending the influence of age and gender on AIP levels and should be considered when assessing the cardiovascular health profiles of patients.

IR is considered a factor connecting impaired carbohydrate metabolism to a higher risk of CVD^17^. IR, a possible cause of T2DM, is significantly and positively correlated with AIP, as indicated in previous studies^18^. In an Australian cohort study with 161 participants, AIP was significantly higher in men and patients with T2DM (p=0.009)^19^. Akbaş et al. found that HbA1c has the strongest correlation with AIP and stated that high AIP levels may be indicative of prediabetes, diabetes, and possibly CAD^20^. Similarly, a study of 4,252 patients reported a parallel increase in AIP with an increase in HbA1c^7^. Li et al. found that AIP was associated with HOMA-IR, HbA1c, and FBG and that metabolic syndrome had a significantly higher prevalence in the group with high AIP^21^. Similar to these studies, we observed a significant positive association between AIP and HOMA-IR. This suggests that AIP could serve as a valuable marker for assessing IR, prediabetes, and the potential development of diabetes.

Higher levels of NLR and PLR have been shown to correlate with the severity of inflammation. Researchers have found that NLR and PLR are closely associated with T2DM and its microvascular complications^22^. Allahverdiyev et al. studied patients with myocardial infarction and found that the NLR and PLR values of patients in the high AIP group were lower than those in the group low AIP^23^. A study conducted on patients with chronic kidney disease found a positive correlation between AIP and NLR and PLR, with AIP also associated with CVD^24^. We found that AIP is correlated with neutrophil and lymphocyte values, and a significant negative correlation exists between AIP and PLR. PLR has been observed to increase in various inflammatory conditions such as thyroid disorders, gastrointestinal diseases, thyroiditis, cancer, diabetes mellitus, and irritable bowel disease^25^. This finding suggests that AIP not only correlates with cardiovascular risk but also exhibits a strong association with inflammation. Similarly, our findings revealed a noteworthy positive correlation between AIP and CRP, indicating a potential relationship between AIP and inflammatory processes. This observation supports the notion that AIP could be a valuable marker for assessing inflammatory conditions.

This study had limitations. Since this study included a limited number of patients, larger studies are required for more comprehensive results. Conducted at a single center in Turkey, the findings may not be widely applicable, necessitating validation through larger, multicenter studies for broader relevance. In addition, only baseline AIP data were available, preventing analysis of changes over time and their impact on outcomes.

In conclusion, we found strong relationships between AIP and both HOMA-IR and PLR. AIP is a promising predictor of IR and T2DM, with strong links to inflammation. AIP is easily measurable and valuable for diagnosing and monitoring diabetes and inflammation. Lowering AIP by correcting lipid abnormalities may reduce the risk of IR and T2DM, making it a useful tool in routine clinical practice for early risk assessment and management.
